# Editorial: Personalized nutrition and gut microbiota: current and future directions

**DOI:** 10.3389/fnut.2024.1375157

**Published:** 2024-02-29

**Authors:** Giuseppe Iacomino, José Ángel Rufián Henares, Fabio Lauria

**Affiliations:** ^1^Institute of Food Sciences, National Research Council (CNR), Avellino, Italy; ^2^Departamento de Nutrición y Bromatología, Instituto de Nutrición y Tecnología de los Alimentos, Centro de Investigación Biomédica, Universidad de Granada, Granada, Spain; ^3^Instituto de Investigación Biosanitaria, Universidad de Granada, Granada, Spain

**Keywords:** gut microbiota, personalized nutrition, dysbiosis, diet, gut microbiome

The community of microorganisms inhabiting the gut, collectively known as the gut microbiota, has emerged as a crucial player in orchestrating multifaceted interactions with various organs beyond the gut itself. Through intricate communication with endocrine, humoral, immunological, metabolic, and neural pathways, the gut microbiota exerts a profound influence on fundamental physiological processes and an individual's susceptibility to various diseases. This intricate community, teeming with trillions of microorganisms, harbors over 100 bacterial species, which possess about 150 times more genes than the human genome. Of note, the microbiota composition has revealed high variability across different anatomical sites, and among these, the gut microbiota stands out with particular significance in safeguarding host health.

While bacteria predominantly constitute the gut microbiota, it also includes protozoa, viruses, archaea, and fungi. Typically, the gut microbiota comprises six phyla: *Actinomycetota* (past *Actinobateria*), *Pseudomonadota* (past *Proteobacteria*), *Fusobacteriota* (past *Fusobateria*), and *Verrucomicrobiota* (past *Verrucomicrobia*), with *Bacillota* (past *Firmicutes*) and *Bacteroidota* (past *Bacteroidetes*) being the predominant types. Besides, the gut mycobiota include *Candida, Saccharomyces, Malassezia*, and *Cladosporium* (1). Given the significant inter-individual variations in gut microbiota composition, it is challenging to establish a definitive definition of a “normal” microbiota (2). Interestingly, the gut microbiome is a dynamic ecosystem that undergoes continuous shifts throughout life. As discussed in the review by Matute and Iyavoo, this dynamism is influenced by a myriad of factors, including environmental cues and host-specific characteristics such as genetics and age. Determinants such as diet, physiological status, exercise, drugs, systemic disorders, and infections can also bring about changes in its composition.

The gut microbiome plays a crucial role in maintaining overall health by orchestrating a symphony of essential functions from maintaining intestinal integrity to generating mucus, promoting regeneration of the intestinal epithelium, fermenting food and producing bioactive metabolites such as those from phenolic compounds, synthesizing vitamins, stimulating the immune response, and defending against pathogens. Besides, it plays a role in the maturation of the innate immune system and acts as a mediator for processing and responding to signals from the environment throughout the body. With recent advances in sequencing technologies, microbiota research has focused primarily on examining the connection between changes in microbiota composition and different health conditions. Population-based studies demonstrated the association of gut dysbiosis with a variety of human diseases, including inflammatory, metabolic, cardiovascular, hepatic, neurological, urinary, respiratory, and skin conditions, and several types of cancer (Figure 1) (3, 4). Accordingly, the study from Venugopal et al. identified specific gut microbiome signatures associated with neutrophil to lymphocytes ratio and mean corpuscular volume in anemic and non-anemic Odisha's low-middle-income rural residents. Besides, the causal connection between dietary changes and therapeutic benefits observed in various clinical settings is increasingly recognized by the scientific community. However, comprehension of the underlying mechanisms by which the gut microbial community exerts its positive or detrimental effects remains largely undetermined. Understanding the factors that influence gut microbiome composition is crucial for developing strategies to promote a healthy and diverse gut microbiota, which can contribute to overall wellbeing and protect against various health issues (5).

**Figure 1:**
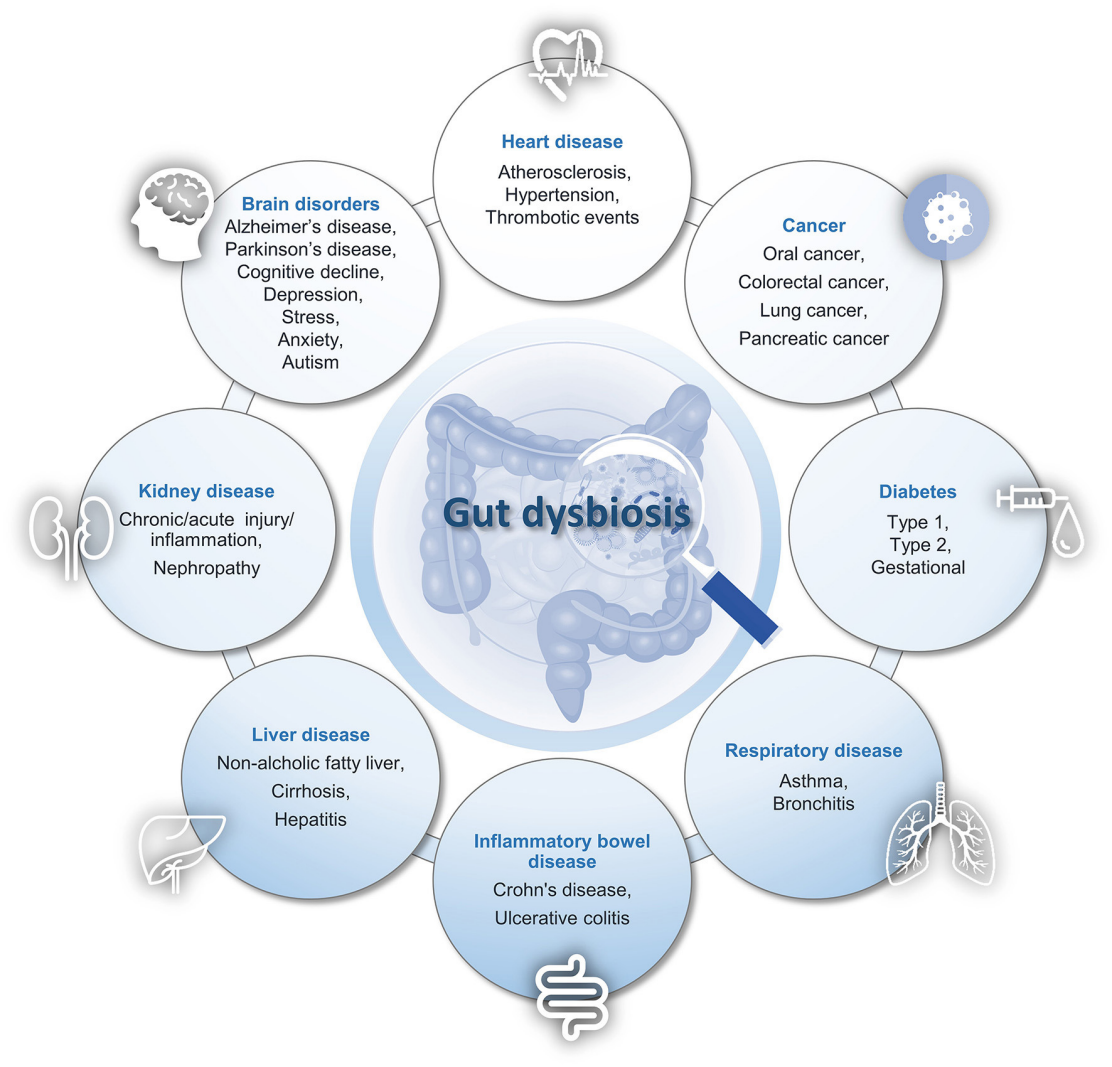
Gut Microbiota dysbiosis in human disease: a partial list.

While the initial phases of life and the genetic traits of the host significantly affect the gut microbiota, it remains adaptable and can be influenced by exposure to diverse environmental factors, including diet (6). Accordingly, the microorganisms that make up the intestinal microbiota exhibit unique responses based on the type and quantity of food. In fact, dietary components influence the abundance of diverse microbial species inhabiting the gut, which in turn generate essential metabolites and signals that exert regulatory control over the host's health status (7).

Besides, the impact of a specific diet can be shaped by a combination of host and microbiome characteristics (8). For example, consumption of exclusively animal or plant-based diets prompted changes in the gut microbiota within 4 days, with fat or fiber alterations evident within 2 weeks (9). Minor nutritional adjustments had minimal impact. Other studies revealed that a diet rich in animal proteins consistently increased *Bacteroides* levels and reduced saccharolytic microorganisms. Interestingly, plant-based protein diets increased lactobacilli and bifidobacteria probiotic components of the gut microbiota. Yet, dietary fiber from fruits, vegetables, and whole grains maintains a thriving microbial ecosystem by promoting short-chain fatty acids (SFCA) production and enhancing immune defenses (10). Conversely, a high intake of saturated fats fosters a pro-inflammatory microbiota, leading to increased intestinal permeability and circulating lipopolysaccharides, potentially contributing to chronic diseases like obesity in both children and adults (11).

To date, only a limited number of studies have delved into precision nutrition approaches, integrating microbiome composition, physiological and metabolic parameters to tailor dietary plans based on individual gut microbiota composition. In the research topic “Personalized nutrition and gut microbiota,” important dietary strategies that focus on providing personalized suggestions are addressed. In the study of Barber et al., the effects of continuous guar gum consumption on selective adaptation of intestinal microbiota taxonomy and metabolic functions that may confer host health benefits were discussed. Besides, as reported in the article by Pagliai et al., the administration of *Lactiplantibacillus plantarum* IMC 510^®^, a probiotic with the ability to enhance the growth of beneficial gut bacteria and alleviate waist circumference, fasting glucose levels, and digestive issues in obese individuals may be a promising approach for obesity prevention and management.

In this expanding field, present-day nutritional guidelines for a healthy diet neglect crucial aspects related to the gut microbiome, such as the impact of specific dietary components on microbial diversity, the potential modulation of the gut microbiota through probiotics and prebiotics, and the necessity for personalized microbiome analysis to accommodate individual variations in microbial composition. However, modulating a correct diet aiming at shaping a “healthy gut microbiota” remains a challenging task. The analysis of the human gut microbiome is still at an early stage to fill knowledge gaps about the community dynamics, microbiome-host relationship, its role in disease pathogenesis, and its therapeutic implications. More in-depth research is needed to unveil this fascinating and enigmatic area of study.

## Author contributions

GI: Writing—original draft, Writing—review & editing. JR: Writing—original draft, Writing—review & editing. FL: Writing—original draft, Writing—review & editing.
